# Intraoperative Assessment of Margin Accuracy in Early Oral Squamous Cell Carcinoma (cT1, T2, N0): Clinical Versus Frozen Section Analysis

**DOI:** 10.7759/cureus.36699

**Published:** 2023-03-26

**Authors:** Tanmay P Tapase, Rajat K Patra, Deepak K Kisku, Samit K Badhai, Sudhir K Panigrahi, Gaurav Bhoopathy, Prasanna K Debata

**Affiliations:** 1 Department of General Surgery, Kalinga Institute of Medical Sciences, Bhubaneswar, IND

**Keywords:** oral squamous cell carcinoma, surgical oncology, margin assessment, intraoperative frozen section, clinical assessment

## Abstract

Background

The incidence of oral cavity cancer is increasing. During oral carcinoma surgery, to achieve a tumor-free margin, intraoperative margin assessment includes two primary methods, namely, clinical examination and frozen section analysis. With extensive preoperative imaging investigations and intraoperative clinical margin assessment, the need for further cost and resource-intensive frozen section analysis has recently come under question. This study aimed to assess whether frozen section analysis can be safely omitted in most cases of early oral squamous cell carcinoma surgeries for cost-effectiveness.

Methodology

A hospital-based, observational study including 30 admitted cases of early oral squamous cell carcinoma was conducted at the Department of General Surgery, Pradyumna Bal Memorial Hospital, Bhubaneswar. All consecutive confirmed cases of early oral squamous cell carcinoma of all age groups and both genders after considering the inclusion and exclusion criteria were involved in the study. A comparative assessment of the free margins after tumor excision was done by the surgeon followed by frozen section analysis.

Results

The mean age was 53.03 ± 13.72 years, with a male-to-female ratio of 6.5:1. Carcinoma of the lower alveolus with gingivobuccal sulcus was the most common presentation of the study (33.33%). In our study, clinically assessed margins had a sensitivity of 75.39%, a specificity of 94.43%, and an accuracy of 92.77%. Frozen section assessed margins had a sensitivity of 66.5%, a specificity of 96.94%, and an accuracy of 92.77%.

Conclusions

Based on the accuracy of clinically assessed and frozen section assessed margins, this study concluded that surgically resected/excised specimen by the surgeon plays a vital role in assessing the adequacy of resected/excised margins in early oral squamous cell carcinoma (cT1, T2, N0) cases, which can possibly replace the costly frozen section analysis.

## Introduction

Oral cavity cancer is a relatively uncommon disease globally, whereas, in the subcontinent of India, it has a disproportionately higher incidence rate. Current studies suggest that the incidence of oral cavity cancer is on the rise [1-3]. With the larger disease burden in southern Asia, it is imperative to study the incidence, diagnosis, and, most importantly, treatment modalities of oral cavity cancer [4-6]. Management of oral cavity cancer is multidisciplinary at present with the involvement of onco-surgeons, pathologists, radiologists, and other related physicians. Despite this, surgical excision is the main modality of treatment along with radiotherapy with or without chemotherapy recommended postoperatively in cases with evidence of adverse pathological features [7,8]. Wide local excision is a procedure in which the tumor is resected along a fringe of the circumferential normal tissue. This boundary of normal tissue is called the margin of excision specimen and is critical in determining if the entire tumor has been resected or if there is a need to revise the resection margin [9].

As in the case of wide local excision, a primary part of the operative procedure is to get a tumor-free margin that would ensure that the entire tumor has been successfully excised. This mandates a thorough and proper intraoperative assessment of the margin of the excision specimen. Intraoperative margin assessment includes two primary methods, namely, clinical examination and frozen section analysis [10]. Of all the treatment modalities, the impact of surgery is much bigger compared to adjuvant chemo or radiotherapy in the prognosis of positive margins [11].

With extensive preoperative imaging investigations and intraoperative clinical margin assessment, the need for further cost and resource-intensive frozen section analysis has come under question [12].

This study aimed to assess the adequacy of margins for wide local excision of early squamous cell carcinoma (cT1, T2, N0) by clinical assessment or frozen section and determine whether frozen section can be safely omitted in a maximum number of cases of oral squamous cell carcinoma surgeries for cost-effectiveness.

## Materials and methods

A hospital-based, prospective, observational study was conducted at the Department of Surgery, Pradyumnabal Memorial Hospital, Kalinga Institute of Medical Sciences from 2020 to 2022 after ethical approval (approval number: KIIT/ KIMS/ IEC/ 470/ 2020). According to the inclusion criteria, all biopsy-confirmed early oral squamous cell carcinoma (cT1, T2, N0) cases aged >15 years were included in this study. Immunosuppressive patients and advanced stages of oral malignancies were excluded from the study.

A detailed history of biopsy-confirmed early oral squamous cell carcinoma was recorded. Routine blood investigations such as complete blood count, random blood sugar, blood urea and serum creatinine, serum electrolyte, and viral markers (human immunodeficiency virus, hepatitis B surface antigen, and hepatitis C virus) were conducted. Ultrasonography of the neck, CT of the neck, high-resolution CT of the thorax, and MRI of the brain were obtained before surgery. Preoperative informed consent was taken, and the possible complications were explained to the patient and relatives. Frozen section analysis of the margins of excised tumors was done and compared with the surgeon’s assessed margins.

Margins of the resected specimen were demarcated and labeled by tying a short thread on the superior margin and a long thread on the lateral margin, followed by assessment of margins by frozen section analysis using a cryostat machine (Minux®FS800A). Margins that were reported positive or suspected on frozen section analysis were revised until clear margins were reported. The percentage of clinically assessed margins that were free from the tumor on frozen section analysis and reconfirmed with final histopathology were noted. The percentage of margins which were reported as positive or suspected on frozen section analysis and found to be positive on final histopathology were noted. The percentage of margins which were reported as free from the tumor on frozen section analysis but found positive in the final histological examination were also noted.

Statistical analysis was done using SPSS version 23.0 (IBM Corp., Armonk, NY, USA). Categorical variables such as age, gender, and site of the lesion were expressed with their mean and standard deviation. Assessment of both surgical expertise and the frozen section of the free margins of the carcinomatous lesion was done through sensitivity and specificity analysis, and positive predictive value, negative predictive value, and accuracy were determined by the standard statistical formula. P-values <0.05 were considered significant.

## Results

Age and gender variation

A total of 30 patients underwent the surgery. The mean age of the patients was 53.03 ± 13.72 years. The maximum age in the study was 75 years, and the minimum age was 33 years. Regarding the gender distribution, the male-to-female ratio was 6:1. The total percentage of male participation was 86.67% while female participation was 13.33%.

Type of lesion

The most common presentation was carcinoma of the lower buccal mucosa with involvement of the lower alveolus and gingiva-buccal sulcus (39.99%), with the second most common presentation was carcinoma of the left buccal mucosa (20%) (Table 1).

**Table 1:** Location of oral squamous cell carcinoma.

Diagnosis	Frequency	Percentage	Cumulative
Carcinoma of the left lower gingiva-buccal sulcus	1	3.33	3.33
Carcinoma of the right lower gingiva-buccal sulcus	1	3.33	6.67
Carcinoma of the upper palate	1	3.33	10.00
Carcinoma of the right buccal mucosa	2	6.67	16.67
Carcinoma of the right retromolar trigone	2	6.67	23.33
Carcinoma of the tongue left lateral	2	6.67	30.00
Carcinoma of the left buccal ulcerative mucosa	3	10.0	40.00
Carcinoma of the left upper alveolus	4	13.33	53.33
Carcinoma of the left lower alveolus	10	33.33	86.66
Leukoplakia of buccal mucosa	1	3.33	89.99
Carcinoma of the left buccal mucosa	1	3.33	93.33
Recurrent carcinoma of left buccal mucosa	1	3.33	96.66
Right submandibular mass	1	3.33	100

Type of surgery

Wide local excision with segmental mandibulectomy with modified neck dissection III was the most common procedure done during the study (26.66%) while the second most common procedure was wide local excision with supra-omohyoid neck dissection(20%). The least common procedure during the study was wide local excision with hemi-mandibulectomy with modified neck dissection III (6.66%) (Table 2).

**Table 2:** Frequency of the surgery procedures performed for oral squamous cell carcinoma based on different locations.

Surgery	Frequency	Percentage	Cumulation
Wide local excision	3	10.00	10.00
Wide local excision + upper alveolectomy + modified neck dissection III	3	10.00	20.00
Wide local excision + marginal mandibulectomy + modified neck dissection III	5	16.66	36.66
Wide local excision + hemi-mandibulectomy + modified neck dissection III	2	6.66	43.32
Wide local excision + segmental mandibulectomy + modified neck dissection III	8	26.66	70.00
Wide local excision + partial maxillectomy + modified neck dissection III	3	10.00	80.00
Wide local excision + supra-omohyoid neck dissection	6	20.00	100.00

Margin assessment

**Figure 1:**
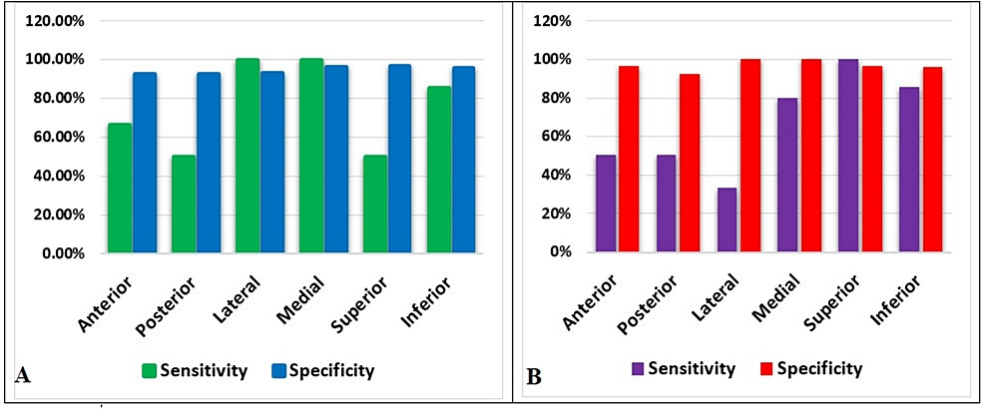
Sensitivity and specificity of margin assessment of both clinical procedure (A) and frozen section (B) in oral squamous cell carcinoma.

Various margins of the lesion were analyzed in the study such as anterior, posterior, lateral, medial, superior, and inferior margins. All margins were analyzed by the oncology surgeon and subjected to frozen section analysis of the free margins (Figure 1, Table 3).

**Table 3:** Sensitivity, specificity, and accuracy of both clinical and frozen section analysis. NPV: negative predictive value; PPV: positive predictive value

Margin	Clinically assessed margin					Frozen section assessed margin				
	Sensitivity	Specificity	PPV	NPV	Accuracy	Sensitivity	Specificity	PPV	NPV	Accuracy
Anterior	66.66%	92.59%	66.6%	92.5%	90%	50%	96.15%	50%	96.15%	90%
Posterior	50%	92.59%	50%	92.59%	86.66%	50%	92.30%	50%	92.30%	86.66%
Lateral	100%	93.10%	100%	93.10%	93.33%	33.3%	100%	33.3%	100%	93.33%
Medial	100%	96.15%	100%	96.15%	96.66%	80%	100%	80%	100%	96.66%
Superior	50%	96.55%	50%	96.55%	96.66%	100%	96.55%	100%	96.55%	96.66%
Inferior	85.71%	95.65%	85.71%	95.65%	93.33	85.71%	95.65%	85.71%	95.65%	93.33

## Discussion

In this study, the mean age was 53.03 years which is comparable with other studies by Wasif et al. and Demir et al. where the mean age of presentation was 52 and 57.4 years, respectively [13,14]. Regarding gender distribution, this study included more males (86%) than females (13%) which is comparable with other studies by Wasif et al. [13]. The higher incidence in males was because of poor oral hygiene and constant use of betel which irritates the lower buccal mucosa and the alveolus causing cancer in the lower gingivobuccal sulcus. Oral carcinoma in western countries is due to unequal sharpness of teeth which can cause continuous irritation to the mucosa of the tongue [13-15].

Location of the lesion

The most common location of the lesion in this study was the lower buccal mucosa with involvement of the gingivobuccal sulcus while other studies reported other common locations of oral carcinoma. The most common site of oral cancer was reported to be the lateral aspect of the tongue in the studies by Demir et al. [14] and Abbas et al. [15] while the most common site of cancer in the study by Wasif et al. was the buccal mucosa [13].

Accuracy of the frozen section assessment

All margins that showed tumor involvement or were inadequate on the frozen section were revised until adequate or negative margins were achieved. Margins in all six dimensions of anterior, posterior, superior, inferior, medial, and lateral were exposed for the frozen section analysis on the paraffin-embedded section. A comparison of various studies of frozen section assessment was done. The accuracy of the frozen section in this study was 92.77% while the accuracy in other studies by Wasim et al., Demir et al., and Abbas et al. was 99%, 96%, and 90.09%, respectively [13-15]. The frozen section accuracy of this study was in the same range as in previously reported studies.

The cost-benefit of the frozen section analysis was assessed in this study. Most studies reported that a frozen section cannot be beneficial in controlling loco-regional metastasis but the frozen section is useful for planning adjuvant therapy for patients. Though the cost of the frozen section is comparatively very less compared to the western countries with a very low cost-benefit ratio (12.2:1). Studies by DiNardo et al. have proved the cost-benefit ratio of frozen section as 20:1, the method was not acceptable as they performed a cohort study in 80 patients [16,17].

As the frozen section is costly and the cost-benefit ratio is also low, the alternative is the free margin assessment by the surgeon on the table. Revision surgery can be done if the resected margins do not have 7 mm free tumor margins to achieve 7 mm tumor-free margins. In this study, six out of eight cases were identified in which margins were inadequate on frozen section analysis due to the microscopic involvement of the lesion. The use of the 7 mm criteria along with gross examination can single-handedly report inadequate margins in eight out of eight cases. Hence, margin revision could have been done only based on gross examination by an expert onco-surgeon. Moreover, the frozen section missed inadequate margins in two cases which were grossly detected by the surgeon intraoperatively. Hence, it can be stated that the identification of inadequate margins can be done by gross clinical examination in the majority of cases [17].

The limitation of the study was the low sample size for a clear comparison between clinical margin assessment and frozen section margin assessment.

## Conclusions

Early oral squamous cell carcinoma (cT1, T2, N0) cases were majorly observed in the middle age group (50-60-year age group). The most common site of oral carcinoma is the lower alveolus and gingivobuccal sulcus. The sensitivity, specificity, positive predictive value, negative predictive value, and accuracy of clinical assessment and frozen section are almost similar to each other. Hence, it can be concluded that a very meticulous gross examination of the surgically resected/excised specimen by the surgeon plays an important role in assessing the adequacy of resected/excised margins in cases of early oral squamous cell carcinoma (cT1, T2, N0), which can replace the expensive frozen section analysis.
